# Using plant-based compounds as preservatives for meat products: A review

**DOI:** 10.1016/j.heliyon.2023.e17071

**Published:** 2023-06-10

**Authors:** Gabriel Olvera-Aguirre, Ángel Trinidad Piñeiro-Vázquez, José Roberto Sanginés-García, Adriana Sánchez Zárate, Angélica Alejandra Ochoa-Flores, Maira Rubi Segura-Campos, Einar Vargas-Bello-Pérez, Alfonso Juventino Chay-Canul

**Affiliations:** a Instituto Tecnológico de México, Campus Conkal, CP, 97345, Conkal, Yucatán, Mexico; b División Académica de Ciencias Agropecuarias, Universidad Juárez Autónoma de Tabasco, Km 25. Carretera Villahermosa-Teapa, R/A La Huasteca, CP, 86280, Colonia Centro, Tabasco, Mexico; c Facultad de Ingeniería Química, Universidad Autónoma de Yucatán, Periférico Norte Km. 33.5, Colonia Chuburná de Hidalgo Inn, Mérida, Yucatán, Mexico; d Department of Animal Sciences, School of Agriculture, Policy and Development, University of Reading, P.O. Box 237, Earley Gate, Reading, RG6 6EU, UK; e Facultad de Zootecnia y Ecología, Universidad Autónoma de Chihuahua, Chihuahua, 31453, Mexico

**Keywords:** Phenolic compounds, Plant extract, Shelf life, Natural additives, Auto-oxidation

## Abstract

The susceptibility of meat and meat products (MP) to oxidation and microbial deterioration poses a risk to the nutritional quality, safety, and shelf life of the product. This analysis provides a brief overview of how bioactive compounds (BC) impact meat and MP preservation, and how they can be utilized for preservation purposes. The use of BC, particularly plant-based antioxidants, can reduce the rate of auto-oxidation and microbial growth, thereby extending the shelf life of MP. These BC include polyphenols, flavonoids, tannins, terpenes, alkaloids, saponins, and coumarins, which have antioxidant and antimicrobial properties. Bioactive compounds can act as preservatives and improve the sensory and physicochemical properties of MP when added under appropriate conditions and concentrations. However, the inappropriate extraction, concentration, or addition of BC can also lead to undesired effects. Nonetheless, BC have not been associated with chronic-degenerative diseases and are considered safe for human consumption. MP auto-oxidation leads to the generation of reactive oxygen species, biogenic amines, malonaldehyde (MDA), and metmyoglobin oxidation products, which are detrimental to human health. The addition of BC at a concentration ranging from 0.025 to 2.5% (w/w in powdered or v/w in oil or liquid extracts) can act as a preservative, improving color, texture, and shelf life. The combination of BC with other techniques, such as encapsulation and the use of intelligent films, can further extend the shelf life of MP. In the future, it will be necessary to examine the phytochemical profile of plants that have been used in traditional medicine and cooking for generations to determine their feasibility in MP preservation.

## 1 Introduction

The combination of water, high-quality proteins, polyunsaturated fatty acids, carbs, vitamins, minerals, and colors make meat a highly nutritious food source. However, its nutrient composition also makes it susceptible to spoilage [[1], [2], [3], [4], [5], [6]]. The most significant quality loss in meat and MP occurs due to auto-oxidation and microbial deterioration [[7], [8], [9], [10]]. These processes lead to the formation of undesirable flavors, odors, and toxic compounds, such as reactive oxygen species (ROS) and nitrogen (RNS), that can cause mutagenesis, thus posing a risk to human health [[11], [12], [13]].

The application of preservatives is one way to protect the fatty acids, proteins, and vitamins in MP from auto-oxidation and spoilage [9,11].

By incorporating synthetic antioxidants into MP, such as butyl-hydroxy-anisole (BHA), sulphur dioxide, butyl-hydroxy-toluene (BHT), tert-butyl-hydroquinone (TBHQ), propyl-gallate (PG), nitrites, nitrates, ascorbates, monosodium glutamate, and liquid smoke, the rate of lipid (LA) and protein (PA) auto-oxidation can be slowed down. This lengthens the shelf-life of the product and prevents spoilage [[14], [15], [16], [17], [18]].

Despite their effectiveness, it has been confirmed that these preservatives can cause food poisoning, liver damage, carcinogenesis, and mutagenesis [12,14,17,[19], [20], [21]].

Because of the potential health risks associated with synthetic preservatives, the meat industry is looking for BC as a safer alternative. One such form of BC is essential oils (EO) or plant extracts (PE), which have been found to have antioxidant and antimicrobial properties [[22], [23], [24]]. The use of BC rather than synthetic antioxidants is considered safer, easier to apply, and more sought after by health-conscious consumers [13,[25], [26], [27], [28]].

Plant extracts and EO are the main natural sources of BC that are used in MP. These compounds are defined as molecules of phenolic acids (such as hidroxytirosol and tirosol), flavonoids, tannins, terpenes, catechins, alkaloids, saponins (such as dihydropyranones), and coumarins that act as antioxidants and antimicrobials [12,25,28,29].

Studies have shown that these natural compounds can be effective against various microorganisms (MO) such as *Bacillus anthracis* [30], *Salmonella typhimurium* [30,31], *Pseudomonas aeruginosa* [24], *Escherichia coli* O157:H7 [[30], [31], [32]], *Listeria monocytogenes* [30], *Staphylococcus aureus* [33], *Bacillus fastidiosus*, and *Salmonella choleraesuis*, among others [30,32,[34], [35], [36]]. Although BC are generally deemed safe for human consumption, it is still important to conduct toxicity studies before introducing them to the food market.

The composition of meat can cause rapid LA and PA, leading to a shorter shelf-life and poor quality proteins, resulting in economic losses for the meat industry [17,37]. In industrialized countries, there is a demand for healthier, high-quality proteins with a longer shelf-life [21]. By utilizing BC such as PE and EO, the meat industry can naturally extend the shelf-life of MP while improving their quality and safety. This can lead to economic benefits for the industry while meeting the needs of health-conscious consumers who demand high-quality, natural food products.

Bioactive compounds from medicinal plants and spices can be utilized in various forms such as PE, EO, oleoresins, powders, fruits, stems, and flowers, and at different doses ranging from 0.025% up to 2.5% w/w, w/v, v/v [13,25,[38], [39], [40]]. Although much research has already been done on the use of BC in the meat industry, there are still gaps in knowledge regarding the utilization of natural compounds to replace or reduce synthetic antioxidants.

One of the biggest challenges in the meat industry is extending the shelf-life of MP without compromising their quality and safety. Incorporating BC can improve the nutritional value, taste, oxidative stability, and protect consumers from free radicals that can cause chronic diseases. Therefore, this review aims to provide discuss how BC impact the meat and MP preservation, and how they can be utilized for preservation purposes. By examining the existing research on this topic, we hope to provide a better understanding of the benefits and limitations of using BC in the meat industry.

## 2 Biochemistry of lipid and protein oxidation in MP

Meat contains a high number of oxidizing agents. When meat becomes rancid, appears discolored, loses water and nutrients, and forms toxic compounds from the denaturation of the lipids and proteins, it has become oxidized and is no longer safe to eat [26,40].

Oxidation in food is known as auto-oxidation since it occurs *in situ*. It is the loss of at least one electron from meat and MP exposed to oxygen during handling and is the most important cause of non-microbial quality loss [10,26]. During lipid oxidation in meat and MP, degradation of unsaturated fatty acids occurs, forming aldehydes, ketones, and alcohols that confer the reheated flavor characterized by a cardboard-like, and rancid-odor taste [17].

**Fig. 1:**
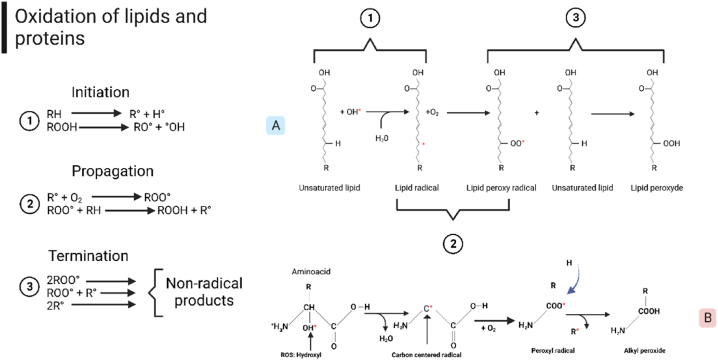
The chain reaction oxidation that includes three phases: (1) reaction initiation, (2) propagation and (3) termination. Adapted from Refs. [26,42,86]. Created with BioRender.com [88].

One of the main aldehydes generated during secondary lipid oxidation is MDA, which is used to measure the oxidation process of lipids [19]. Lipid auto-oxidation is described in Fig. 1 (A), which is a chain of three processes that include initiation, propagation, and termination reactions. Undesirable flavors and odors are attributed to the production of volatile compounds of radical fatty acids (such as superoxide anion, hydroxyl [LOH], peroxy [LOO], hydroperoxy [HO2], lipid radical [RO], nitric oxide [LNO], nitrosyl cation [LONH_2_], and hydroperoxides [LOOH]) [17,41,42].

Proteins and amino acids are another medium where oxidation reactions occur (Fig. 1 B). These reactions generate cytotoxic substances such as ROS (peroxyl radicals [LOO], alkyl peroxide [LOOH], carboxyl [LCOOH], alkoxyl [LO], hydroxyl [LOH]), as well as biogenic amines and their precursors (histamine, cadaverine, tyramine, putrecine, tryptamine, histidine, lysine, tyrosine, ornithine and tryptophan) [43].

Guedes-Oliveira et al. [41] reported an example of lamb hamburger meat treated with 100 mg/kg of *Myrciaria dubia* leaf and seed extract for 9 days at 4 °C not successfully delaying the PA.

These treatments exerted a pro-oxidant effect on the protein-liposome system and did not stop the formation of protein carbonyls.

Huang et al. [44] observed PA by evaluating the production of eight biogenic amines (trypamine, phenylethylamine, putrecine, cadaverine, histamine, tyramine, spermidine and esperimine) in refrigerated carp fillet (4 °C for 12 days) treated with cinnamon bark oil (0.1% v/v). They observed cinnamon bark oil delay the increase of putrecine and cadaverine and MO from the control, but not in the other amines studied. This is attributed to the antimicrobial effect due to cinnamaldehyde of cinnamon oil on some bacteria (*Aeromonas* and *Shewanella*) that produce biogenic amines.

Cinnamaldehyde is the main BC of cinnamon bark oil, which contains between 50 and 90% of this compound, as well as minor concentrations of eugenol, linalool, and alpha-pinene. Cinnamaldehyde has been found to have the ability to inhibit the production of essential enzymes and damage the cell walls of bacteria. However, *Pseudomonas* is highly resistant to cinnamon bark oil as the lipopolysaccharides present in their outer membrane can prevent cinnamaldehyde from effectively penetrating it. Despite this resistance of *Pseudomonas* to cinnamon bark oil, it remains an effective natural antimicrobial agent against many other types of bacteria commonly found in food products.

The color of meat is the first attribute of quality that the customer perceives, and it is mainly conferred by hemoglobin and myoglobin [9]. Myoglobin is a highly reactive sarcoplasmic protein composed of globin with a heme group at its center, which is a prosthetic group responsible for color and binding to other molecules, giving it an oxide-reduction property [45].

When myoglobin is present along with Fe in the reduced state, it generates a purple-red hue. This form of myoglobin is known as deoxymyoglobin. On the other hand, when oxymyoglobin is accompanied by reduced Fe, it produces a bright red color. Metmyoglobin is produced in the presence of oxidized Fe, resulting in a grayish-brown color [46]. During these reactions, intermediate reagents can be generated that have the potential to catalyze the oxidation of unsaturated fatty acids [45,47]. Furthermore, Fe can react with substances such as carbon monoxide, sulfites, or ascorbates, leading to the production of carboxyhemoglobin, sulfimioglobin, and cholemyoglobin, which can manifest as green colors [36].

Meat oxidation damages the texture, smell, flavor, and color, which impacts storage life and generates economic losses and food waste. Therefore, it is important to protect MP against microbial deterioration and oxidation of lipids, proteins and heme groups.

## 3 Quality and shelf life of meat products

The quality of MP is determined by their physicochemical and sensory characteristics such as available nutrients, color, smell, and texture. Loss of these characteristics due to various factors such as lipid oxidation is the main reason for the degradation of MP quality [48]. Traditional preservation techniques such as smoking, curing, fermenting, salting, adding plant extracts, and controlling animal diets have been used to extend the shelf-life of MP. Nevertheless, the shelf-life of MP has been found to be enhanced by emerging techniques such as vacuum packaging, bio-packaging or edible films, incorporating synthetic antioxidants, or a mixture of these approaches [20,[49], [50], [51]]. In particular, retarding lipid oxidation can be improved by combining multiple preservation techniques such as using bioactive films, modified atmospheres, nanoencapsulation, and nanofibers [32,52].

The shelf-life of a MP is defined as the amount of time until the degradation of organoleptic characteristics, leading to quality loss, and product rejection. MP are more susceptible than meat to microbial deterioration, oxidation of lipids, proteins, and pigments, is due to the fact the muscular membranes have already disintegrated due to grinding, and that they have greater contact with surfaces, oxygen, and human manipulation [39]. For these reasons, preservatives are being developed to maintain the quality and shelf life of MP for longer periods [17,53].

Shelf life is measured by color coordinates, microbiology, sensory evaluation, and nutrient oxidation. The proper use of BC as natural antimicrobials added to MP has the effect of prolonging conservation by preventing microbial contamination by pathogens, and reducing antibiotic resistance of MO [23,34].

As one example, Vergara et al. [54] used oregano (*Origanum vulgare*) mixed in the form of oleoresin or EO (at a concentration of 0.08% w/w) and vacuum packaging, and then evaluated the quality characteristics of pH and color coordinates (lightness: L*, red/green: a* × and yellow/blue: b*), along with the shelf life of lamb burgers including LA and MO growth (total viable count, *Enterobacteriaceae*, *Pseudomonas* Spp. and lactic acid bacteria [LAB]). In conclusion, they observed that this method preserved the meat with better color than the control for 14 days, with an acceptable pH of 5.6, and stopped the formation of MAD/kg at the same time.

Oregano has the ability to prevent rancidity and prolong the shelf life due to the presence of phenolic compounds such as luteolin, quercetin, naringenin, and pinocembrin. The antioxidant capacity of oregano plays a crucial role in achieving this effect.

As another example, Cozar [53,55], studied the combined effect of vacuum packaging in combination with spice powder (rosemary, thyme, sage and garlic at a concentration of 0.1% w/w) with lamb burgers, analyzing color coordinates, and MO count (including total viable count, *Pseudomonas* spp, *Enterobacteriaceae* and LAB), and reported that the effect of rosemary (*Salvia rosmarinus*), thyme (*Thymus vulgaris*) and sage (*Salvia officinalis*), was more effective in delaying discoloration and rancidity for 13 days than garlic (*Allium sativum*) and control treatments.

By combining packaging techniques with the addition of plants that are rich in BC such as borneol, verbenone, camphor, alpha-pinene, thymol, terpinene, and linalool, it is possible to maintain the quality characteristics of MP. This approach can effectively help to preserve the quality of meat and extend its shelf life.

When Al-Juhaimi et al. [22] added 2, 4, or 6% of *Moringa oleifera* seed fluor to beef patties and evaluated the results, they found that the shrinkage parameters, including raw and cooked thickness, as well as raw and cooked diameter, decreased as the percentage of moringa increased. However, fat retention, moisture retention, and cooking yield increased proportionally with higher percentages of moringa powder. In addition, the organoleptic characteristics of general acceptability, juiciness, flavor, taste, and tenderness were maintained for up to 21 days, compared to the control group that was acceptable for only seven days.

*Moringa oleifera* is a legume that is rich in high-quality fats and antioxidants with high activity such as ascorbic acid, flavonoids, phenols, and carotenoids. Due to these properties, *M. oleifera* can be effectively used to extend the shelf-life of MP. The antioxidants present in *M. oleifera* play a significant role in reducing oxidative damage by scavenging free radicals, thereby preventing lipid oxidation and protein degradation. In addition, the high-quality fats found in *M. oleifera* can help to inhibit the growth of spoilage microorganisms. Therefore, the use of *M. oleifera* as a food ingredient can be beneficial in extending MP shelf-life.

In a study by Jayathilakan et al. [8], the addition of 250 mg/100 g of powdered cloves (*Syzygium aromaticum*) to lamb, beef, and fresh minced pork resulted in greater antioxidant activity compared to 500 parts per million (ppm) of BHA, PG, ascorbic acid. The study also compared this to the addition of 250 mg/100 g cinnamon per meat, highlighting the superior ability of some clove BC, such as eugenol, in controlling lipid oxidation compared to synthetic antioxidants. Another study by Fadiloğlu and Çoban [56] found that adding 1% of Goji Berry liquid extract (*Lycium barbarum* L.) to smoked meat resulted in extended shelf-life of carp sausages by reducing microbial counts, improving the color and odor of the sausage, and preventing lipid oxidation. This demonstrates the effectiveness of natural additives in improving the quality and extending the shelf-life of MP compared to synthetic antioxidants.

In their study, Al-Juhaimi et al. [22] observed that the addition of up to 6% (w/w) of Moringa seed flour in beef patties resulted in improved cooking yield, shelf-life, aerobic microbial stability, and consumer preference in refrigerated conditions. This indicates that *M. oleifera* seed flour can be used as a natural additive in MP to enhance their quality and extend their shelf-life.

In contrast, Aquilani et al. [31] observed that the addition of fish oil to pork burgers resulted in reduced protein content and water retention during the cooking process compared to the control group. They also found that burgers treated with fish oil had an undesirable color over the L* parameter. These observations indicate that even among BC, there may be specific mechanisms of action that do not necessarily lead to the improvement of meat product quality, but may actually deteriorate it and cause rejection. Therefore, it is important to carefully evaluate the use of different BC and their concentrations in MP to determine their effectiveness in enhancing their quality and extending their shelf life.

## 4 Biochemistry of bioactive compounds

Bioactive compounds used in MP can be obtained from different sources, such as crustaceans (e.g. chitosan), plants (e.g. extracts and essential oils), and microorganisms (e.g. bacteria that generate bacteriocins and organic acids). Among these sources, plant-derived BC are often preferred for use in MP due to their antimicrobial properties against human and plant pathogens, as well as their ability to extend shelf-life [34,[57], [58]].

Plant-derived BC can be obtained through various methods such as extractions, distillations, fermentations, or enzymatic processes. They are considered safe and have been extensively studied for their beneficial effects on human health, making them a desirable choice as a natural additive in MP.

Efficient extraction of bioactive compounds is needed for the food industry. For that, hybrid technologies combine different processes (conventional and non-conventional) [59]. The new methods being used lead us towards more environmentally friendly technologies and less reliance on chemicals. However, it is crucial to ensure that these techniques are safe without affecting consumer acceptability and this needs proper validation.

There is a need for improving laborious methods (soxhlet, hydro distillation, water distillation, maceration, cohobation, heating reflux extraction) to reduce time, and costs, preserve compounds, and reduce environmental disposal problems. For instance, novel green extractions are ultrasound-assisted extraction (UAE), microwave-assisted extraction (MAE), high pressure, pressurized liquid extraction (PLE), negative pressure cavitations-assisted extraction, subcritical water extraction, supercritical fluid extraction, enzyme-assisted extraction, pulsed electric field-assisted extraction, and accelerated solvent extraction. These green techniques are called e3 and include six principles: 1) well-defined sourcing; 2) limiting the organic solvent usage; 3) minimizing consumption of energy; 4) by-product generation with high potential of value addition; 5) lessening the duration of extraction and 6) natural ingredient recovery [60]. Some of limitations of these techniques are that they are expensive and may not be useful at large-scale level [60].

Junsathian et al. [61] studied three diferent extraction methods (ethanol extraction, microwave-assisted extraction and ultrasonic-asissted extraction) over the total phenolic content, total flavonoids content, antioxidant activity (DPPH and FRAP assay) and antimicrobial activity of six Thai edible plant leaf extracts. They found that MAE improved the antioxidant and antimicrobial efficacy of the leaf extracts in comparasion of ethanol extraction and UAE. All the techniques was doing at a ratio of 1:30 g/mL powder/solvent at 25 °C for 60 min, except for MAE, that was for 60 s. Overall, there is a need for comparing the most efficient techniques of extraction with the best antioxidant and antimicrobial activities and with the least and safest amount of solvent.

In this regard, Borges et al. [62] evaluated different extraction techniques (solid-liquid, UAE, Soxhlet and MAE) with different solvents (water, methanol, ethanol, acetone, dichloromethane, and hexane), and they found that ethanol and acetone provided the best extraction of bioactive compounds, and the best antioxidant and antimicrobial activity. Then, Soxhlet and MAE were the best extraction methods to obtain BC and with higher antimicrobial activity.

In their research Garofulic et al. [63] evaluated the effect of MAE, PLE and conventional heat-reflux extraction techniques on *Urtica dioica* leaves as to the antioxidant capacity assesed by ORAC assay. MAE was the best technique for isolation BC (phenolics). Then, PLE had higher exraction of total BC and antioxidant capacity and had antimicrobial activity against *Pseudomonas fragi* and *Campylobacter jejuni*.

The adverse effects resulting from BC consumption are rare [64]; however, it is important to conduct toxicological assessments to eliminate any possible negative effects. In the United States, this evaluation follows the EFSA guidelines, which outline the steps for performing safety assessments of food additives, involving a tiered toxicity testing process. The FDA also follows the guidelines detailed by the Toxicological Principles for the Safety Assessment of Direct Food Additives and Color Additives Used in Food, commonly referred as the Redbook [65]. Within the European Union, EFSA categorizes the assessment process into three tiers. Tier 1 requires minimal data for all substances, while Tier 2 is mandatory for compounds that demonstrate *in vitro* toxicity or genotoxicity in the gastrointestinal system. Finally, Tier 3 should be conducted after Tier 2 if the BC demonstrates bioaccumulation, *in vivo* genotoxicity, and chronic toxicity [66].

As evidenced in Table 1, many plants have been a great source of BC in the form of vegetal material, plant extracts or essential oils, which have promising antimicrobial and antioxidant bioactivity in both *in vitro* and *in vivo* assays. These BC interact with the pH, water, fats, and proteins of MP, which can have implications for their overall quality and shelf-life [[53], [54], [55],^67^]. This is because BC are functional groups of molecules that can destabilize the lipid bilayer of microorganisms, leading to the degeneration of the cell membrane and, eventually, the death of the microorganism [30,31]. This mode of action makes BC a desirable choice for use in MP to improve their safety, quality, and shelf-life, especially in the absence of synthetic preservatives. However, it is important to note that the effectiveness of BC can be influenced by factors such as concentration, pH, temperature, and the type of MO present, among others. Therefore, it is crucial to carefully evaluate the use of different BC and their application in MP to optimize their effectiveness and ensure their safety.

**Table 1:** Main bioactive compounds used in food and action against microorganisms.

Author	Product and effect	Bioactive components and production plant	Inhibited microorganism	Antioxidant effect	Use in food and/or MP, dosage, and effect
[23,89]	Grape by-product extracts	Catechins, epicatechins, gallic acid and procyanidins.	*Staphylococcus aureus, Listeria monocytogenes, Pseudomonas aeruginosa, Escherichia coli* O157:H7,	Less than 3 mg MDA/kg, 0.74 mg MDA/kg chiken for 9 months.	Hamburgers, salami, 2.5% ˄ microbiological stability of salami. **˅** aerobic mesophilic bacteria.
	Antioxidant	*Vitis vinifera*	*Enterobacteriaceae* and *Pseudomonas* sp	Antioxidant activity is due to the ability to sequestrate radicals, chelate metals, and synergize with other antioxidants	In frozzen chicken
[67]	Thyme	Monoterpenes, sesquiterpenes, phenols, alcohols, ethers, aldehydes, and ketones.	Reduction in the population of *Listeria. Monocytogenes*	ND	Fish burgers, chicken breast and low-fat hot dogs' beef, ND **˅** initial count of viable cells.
	Antiviral, antifungal, antitoxigenic	*Thymus vulgaris*			
[30]	Thyme EO	Thymol, paracymene, carvacrol, gamma- terpinene, alpha-pinene, alpha-thujene.	*Staphylococcus aureus, Escherichia coli, Listeria monocytogenes, and Salmonella Typhimurium*	In the DPPH assay, EO were capable of capturing 83.1% of ROS while the encapsuled oil only 5.1%. This occurred because BC interacted with the wall material, prohibiting full release.	Hamburger-like meat product, 1 g/100 g of spray-encapsulated EO is effective in regulating LAB and antimicrobial activity for up to 14 days.
	Antioxidant, antimicrobial	*Thymus vulgaris*			
[35]	Cinnamon	Cinnamaldehyde and Eugenol.	*Bacillus subtilis, Staphylococcu aureus* and *E. coli*	ND	Meat, 0.8, 1.2, 1.6 and 2% **˅** antibacterial activity and maintains the properties of the meat.
	Antimicrobial	*Cinnamomum zeylanicum*			
[32,52]	Nettle aqueous extract	Ascorbic acid, phenylpropanes derived from shikimic acid, caffeic acid, chlorogenic acid, caffeylmalic acid, beta-carotene.	Mesophilic, psychrotrophic and lactic acid bacteria, *Pseudomonas*, *Enterobacteriaceae* count*, E. coli*	Prevents the formation of free radicals during lipid oxidation	250–500 ppm preserve ground beef meat for 14 days at 2 °C
	Antioxidant, antimicrobial, antiviral	*Urtica dioica* L.		500 ppm is more effective than nitrite/nitrate and BHT	Bioactive coatings preserve trout fillets quality for 15 days at 4 °C.
[49]	Quinoa seed and buckwheat fluor Antioxidant, antimicrobial	Polyphenols.	Total viable MO count.	The addition of buckwheat flour to the burgers resulted in reduced lipid oxidation during storage for 120 days	Burgers with 15 and 30% of fluor provides high quality protein to feed, **˅** oil absorption. **˅** MAD production
		*Chenopodium quinoa*			
[81]	Moringa ethanolic-aqueous extract	Galic acid, rutin, vicenin-2, quercetin-3-*O*-glucoside, coumaric acid, b-carotene, N, a-1-rhamnopyranosyl vincosamide.	Total viable count and lactic acid bacteria	Delays lipid oxidation	Ground beef, 0.5 and 1 g/kg of meat. **˅** microbial growth compared with control and BHT.
	Antioxidant, antibacterial	*Moringa oleifera*			
[9]	Algae extract (brown, red, green) Antioxidant, antimicrobial, antiviral	Alginic acid, laminarin and fucoidan, ulvan, carrageenan, xylan, galactan, porphyran, catechins and carotenoids	ND	Reduces the TBARS index	Raw and cooked pork patties, burgers, meat sauces, 0.001–.01%. ˄ MP shelf life, ˄ nutritional properties, promotes functional properties.
[76]	Cinnamon oil	Terpenes, terpenoids, phenols.	*Aeromonas veronii, Acinetobacter johnsonii, Shewanella putrefaciens* and *Pseudomonas jessenii.*	ND	Carp fillets, 0.1% v/v in an emulsion. **˅** the growth of putrecine and cadaverin, ˄ the shelf life by 4 days more than the control
	Antimicrobial	*Cinnamomum zeylanicum*			
[41]	Camu-camu	Anthocyanins, flavonoids, carotenoids, ascorbic acid. *Myrciaria dubai*	ND	Stabilizes lipid free radicals into less reactive forms.	Lamb ground meat, ND. ˄ lipid stability, deactivates free radicals
	Antioxidant			Protects against lipid oxidation	
[28]	Goji Extract	ND	*Staphylococcus aureus*	Reduces lipid peroxidation	Kasakh-style horse meat smoked and cooked, 0.5–1.0%. ˄ texture, smell, flavor, nutrients, oxidative stability.
	Antimicrobial, antioxidant	*Lycium barbarum berry*			

MP: meat products; ˅: reduce; ˄: improve; ND: not described; EO: essential oil; LAB: lactic acid bacteria; MO: microorganism.

### 4.1 Vegetal material

Plants have been utilized for various purposes for thousands of years, including as a source of food, medicine, and food additives in order to impart flavor, color, and aroma, or to extend the shelf-life of perishable foods [24,64]. Given their historical and continued use, it is essential to identify and document the plants and their phytochemical profiles that have the potential for use in preserving and improving the characteristics of MP [11,24,31,32,39,[68], [69], [70]].

The use of BC as additives require the use of safer and non-toxic techniques [71]. Some extractions are difficult to do with traditional organic solvents (acetone, methanol, isopropyl alcohol, dimethyl sulfoxide, dimethylformamide, and pyridine) which commonly induce acute toxicity and carcinogenicity [72,73]. Some solvents such as chloroform is useful extracted terpenoids, flavonoids, fats and oils but it has sedative and carcinogenic property [74].

The traditional use of these plants suggest potential applications in the development of new natural products for the preservation of meat. Overall, understanding the potential of plant-derived BC can aid in the development of more effective and sustainable approaches for meat preservation that align with consumer and industry demands for natural and minimally processed foods.

### 4.2 Essential oils

Essential oils (EO) are natural plant extracts made up of volatile compounds such as terpenes, polyphenols, and fatty acids, which give them a strong odor [64,75]. EO are oily liquids that are obtained through stem and seed distillation and have been used in MP as an attractive alternative to synthetic antioxidants [30,67]. The main BC with high antimicrobial activity contained in EO are terpenes, terpenoids, and phenylpropanoids [76].

However, the antimicrobial activity of BC contained in EO can be influenced by factors such as pH, fats, carbohydrates, proteins, water, salt, antioxidants, preservatives, and extrinsic factors such as temperature, vacuum packaging, and microorganisms [67]. Essential oils have the best antimicrobial activity at pH values of 5–6 and tend to behave in a more hydrophobic way at acidic pH levels, making it easier for them to enter bacterial cells [67,77].

**Fig. 2:**
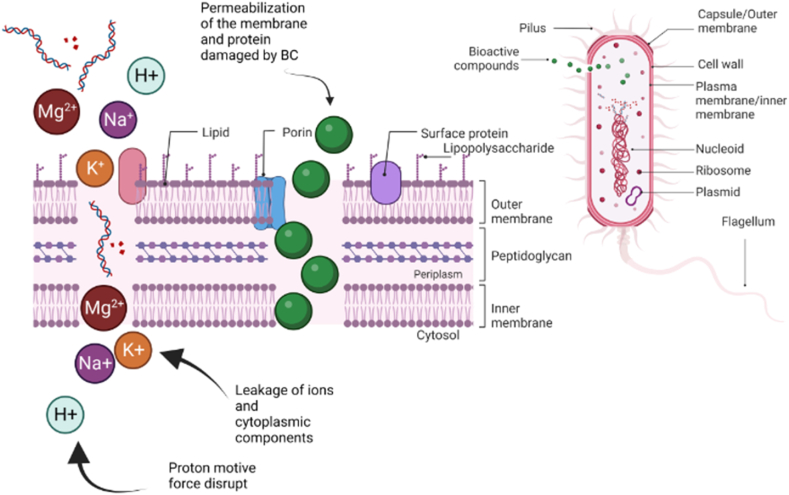
Effect of BC on bacterial cells. Adapted and modified from Hao et al. [76] Created with BioRender.com [88].

Since EO are lipophilic, they can easily enter bacterial cells and permeabilize and/or break through the bacterial cell membrane, as depicted in Fig. 2 [64,78]. When this happens, there is a loss of K^+^, Mg^2+^, and Ca^2+^ ions, which causes a reduction in pH [76]. In eukaryotic cells, EO cause depolarization of the external and mitochondrial membrane, which makes the membrane abnormally permeable [64,67,79]. This can lead to the leakage of radicals, the collapse of the proton pump, and the depletion of ATP, cytochrome *c*, ions, and proteins, ultimately causing apoptosis, necrosis, and cell death [58,68]. In summary, EO have demonstrated promising antimicrobial and antioxidant properties, making them an attractive option for use in meat preservation and enhancement.

Despite the potential of EO for MP preservation, it has been reported that they are highly volatile, which can decrease their effectiveness over time. One solution to this issue is to encapsulate, nanoencapsule EO or incorporate them into slow-release biofilms, which can ensure their optimal use as preservatives. Encapsulation involves enclosing the EO in a protective coating material, such as liposomes or cyclodextrins, which can prevent the EO from evaporating or reacting with other components in the meat product, while also controlling the release rate of the EO [51,80]. Slow-release biofilms, on the other hand, are composite materials that can be used as coatings or packaging films, which release EO over time as the meat product undergoes natural deterioration, thereby extending its shelf-life [50].

When nanoparticles are ingested, they can take different biological pathways in the gastrointestinal system. These particles can be completely absorbed, partially digested and slowly release their encapsulated compounds, resist digestion and be excreted from the digestive system, or go across the intestinal epithelium and enter the bloodstream. Nanoparticles can also absorb gastrointestinal enzymes, which can alter the normal digestion process and accumulate in tissues, leading to toxic effects. Therefore, it is important to conduct thorough toxicological assessments of nanomaterials before they are introduced into the food market, to ensure the safety of the products for consumers. This includes assessing how the particles are absorbed, metabolized, and excreted, as well as evaluating any potential toxicity or harmful effects of the nanoparticles on human health [66].

The use of encapsulation or slow-release biofilms can help reduce the amount of EO needed to effectively preserve the meat product, while also minimizing any potential negative impacts on the sensory properties of the meat product [11,21,30,80]. Additionally, these approaches may also improve the overall safety of the meat product, as the encapsulation or biofilm coating can act as a barrier against harmful microorganisms and as a controlled release system for the EO [30]. Overall, the use of encapsulation or slow-release biofilms offers a promising approach to make the most of the antimicrobial and antioxidant properties of EO for meat preservation.

### 4.3 Plant extracts

Plant extracts (PE) are biological materials that have been washed, dried, ground, and extracted with solvents such as water, ethanol, methanol, a mixture of these, and acetone, among others [24,29,81]. The main compounds in PE can reach concentrations of up to 85%, such as polyphenols like gallic acid, caffeic acid, quercetin, luteolin-7-0-rutinoside, and epigallocatechin, with the remaining compounds being minor components that are essential for synergistic preservation properties [67].

Plant extracts can prevent the formation of free radicals and slow down oxidation, as demonstrated in the case of nettle extracts, which showed greater antioxidant activity in ground beef and lower rates of TBARS compared to synthetic preservatives like BHT, nitrites, nitrates, quercetin, and alpha-tocopherol [32]. This antioxidant effect is attributed to the phenolic compounds, beta-carotene, and vitamin C contained in the nettle plant (*Urtica dioica* L.) [52].

Plant extracts containing BC with high antioxidant and antimicrobial activities that are used to extend the shelf-life of MP. However, it is important to evaluate the most appropriate way to preserve different meat matrices and determine the specific mode of action of the PE to avoid lipid oxidation, protein oxidation, oxidation of myoglobin, or the development of microbial deterioration. By understanding the most effective use of PE in addressing these issues, the meat industry can develop new and innovative approaches to meat preservation that aligns with consumer preferences for natural and minimally processed foods.

### 4.4 Antioxidant activity of bioactive compounds

The most important BC in PE are phenolic compounds, terpenes, and alkaloids [34,82,83]. Phenolic compounds, such as flavonoids, hydroxybenzoic acids, and hydroxycinnamic acids, have a chemical structure that consists of an aromatic ring with one or more hydroxyl groups. These compounds have a relatively simple molecular structure and can also undergo high degrees of polymerization [13,17,31,36].

Flavonoids, phenolic acids and polyphenols have oxidation reducing capacity, as they can donate hydrogen atoms and deactivate singlet (or excited) oxygen, and chelate pro-oxidant metal ions [7,11]. Flavonoids, for example, are widely distributed in plant tissues and vegetables. Hydroxybenzoic acids, such as gallic acid, are also found in plants and are used as a classic reference material to test free radical-scavenging activities [42].

Phenols are polyhydroxy aromatics with the ability to prevent trapping, eliminate free radicals (hydroxyl, peroxyl and superoxide), inhibit lipoxygenase and cyclooxygenase enzymes (causes of rancidity) [11,47]. Hydroxycinnamic acids, such as caffeic acid and ferulic acid, are involved in numerous biological functions related to antioxidant, anti-inflammatory, and antimicrobial activities, as well as cell proliferation and apoptosis [28].

Terpenes, on the other hand, are synthesized from five-carbon building blocks known as isoprenes units of five carbons that may be encountered in either a simple or polymerized molecular structure, and can contain oxygen atoms that confer various chemical functions and are also found in PE [34]. Terpenes are known for their antimicrobial and antioxidant properties, as well as their ability to modulate immune responses and exhibit antitumor activities [31,36].

Finally, alkaloids are a diverse group of nitrogen-containing cyclic organic compounds that can be found in various plants and have been studied for their antimicrobial, anti-inflammatory, and antitumor properties. That are synthesized from amino acids by living organisms such as plants [79]. This group includes compounds with a potential effect on the nervous system. Examples of alkaloids found in PE include quinine, caffeine, and morphine [31,79].

Zeb et al. [42] reported several mechanisms that explain the antioxidant action of BC.

1. Hydrogen atom transfer (HAT): In this mechanism, the antioxidant donates a hydrogen atom to a free radical, stabilizing it and converting itself into a radical. The radical is stabilized, forming neutral species (RH, ROH, or ROOH) while the antioxidant is converted to an antioxidant free radical (A°). This mechanism is mainly observed in phenolic compounds.
2. Single electron transfer (SET): The formation of anion R results in a stable species with an even number of electrons. The cation radical ArOH is also a stable species as it is less reactive. When ArOH° reacts with a free radical, it forms an odd electron that can be distributed over the entire molecule. This arrangement creates an aromatic structure.
3. Single electron transfer-proton loss-electron transfer (SPLET): In this mechanism, the BC donates a proton to a free radical, forming an anion, which then donates an electron, forming a stable molecule. The proton-donating ability and the electron transfer enthalpy are calculated to determine the antioxidant activity. This mechanism is more thermodynamically favorable in water and is thus best suited for phenolic compounds. For diterpenes and coumarins, the polar environment has been reported to favor this mechanism.
4. Transition metal chelation (TMC): In this mechanism, the polyphenols chelate a transition metals (copper, manganese, or cobalt) forming stable products, preventing it from participating in the Fenton reaction. TMC can directly inhibit Fe3+ reduction, consequently reducing the formation of reactive OH-free radicals. Phenolic acids and flavonoids have strong free radical scavenging potential. The ligands of polyphenols strongly stabilize Fe^3+^ over Fe^2+.^ This process is called auto-oxidation [42].

Overall, the antioxidant action of BC is complex and can involve multiple mechanisms depending on the compound and the conditions in which it acts. The BC can offer numerous potential benefits for meat preservation and enhancement. Studies have demonstrated the superior antioxidant efficacy of phenolic compounds found in plant extracts to protect meat lipids better than synthetic antioxidants [46,72].

For instance, the addition of 2% of certain EO antioxidants such as thujone, camphor, alpha and beta pinene from sage (*Salvia officinalis*) and cannabinoids, flavonoids, tetrahydrocannabinol, cannabinol and phenolic acids from cannabis (*Cannabis sativa*) to gelatin coatings on refrigerated pig loin reduced the values of MDA and metmyoglobin (MetMb) compared to the control. Similarly, the addition of sage and cannabis (1:1%) to meat resulted in the highest color stability and the lowest weight loss after 12 days due to their antioxidant activity, which reduced lipid oxidation and thus slowed down LA and PA in pork [47].

In another study, walnut leaf powder containing phenols showed lower LA compared to the control and BHT in chicken burgers [84]. These findings suggest that alkaloids and phenolic compounds found in plant extracts offer excellent potential for natural antioxidant intervention in MP.

### 4.5 Antimicrobial activity of BC

Microbial spoilage in MP can result in the production of biogenic amines and volatile organic compounds due to protein decomposition by MO action. Biogenic amines such as inosine and hypoxanthine can produce undesirable putrid and bitter flavors that consumers are likely to reject [76]. Bioactive compounds found in PE have been shown to be effective in controlling the growth of foodborne pathogens, which can help delay the production of biogenic amines and extend the shelf-life of MP [11,67].

Consumers today are increasingly interested in MP that are preserved using natural ingredients, such as plant extracts. The ability of these natural preservatives to delay the production of biogenic amines and other spoilage compounds offers numerous benefits for the meat industry, including longer shelf-life, increased consumer satisfaction, and reduced food waste. As a result, more research has been focused on exploring the capabilities of BC for improving meat quality, safety, and shelf-life.

Bioactive compounds can be used alone or in combination with active packaging technologies to improve the sustainability, safety, quality, and shelf-life of packaged foods. When used in combination, they create physical, chemical, and biological interactions that alter the native environment of the packaged food, resulting in extended shelf-life and improved product quality [10,31,50].

Meat products generally have a shorter shelf-life than whole meat due to their more porous structure resulting from processes such as grinding. This creates an environment that is more favorable for the proliferation of microorganisms, which can lead to spoilage and potential food safety concerns [53,76,84]. The use of BC and active packaging technologies can help to overcome these issues by slowing down the growth of microorganisms and inhibiting the production of spoilage compounds. This can extend the shelf-life of MP, reduce food waste, and improve product safety and quality. In summary, the use of BC alone or in combination with active packaging technologies offers a promising approach to improve the sustainability, safety, quality, and shelf-life of MP.

In a study by Elhadi et al. [85], it was found that untreated chicken burgers have a shelf life of only 4–10 days. However, by adding 100 g/kg of moringa leaf powder to the burgers, the physicochemical composition was improved, with increased levels of fat, protein, and ashes (30 g/kg, 20 g/kg, and 7 g/kg, respectively). This treatment also reduced peroxide production for 12 days and inhibited the growth of total coliforms, *Escherichia coli*, and *Staphylococcus aureus*.

Essential oils are known to be effective in inhibiting the growth of microorganisms in MP due to their lipophilic nature, which increases bacterial cell permeability and leads to the loss of ions and cytoplasmic constituents (Fig. 2), as well as distortions in lipid-protein interaction, interference with ATPase, disruption of proton force and electron flow, and cytoplasmic coagulation [76]. For example, cinnamon EO has been shown to have strong antimicrobial activity against *Pseudomonas putida* found in meat [35]. In addition, aqueous nettle extracts have been found to inhibit the growth of various bacteria (*Pseudomonas aeruginosa*, *Proteus mirabilis*, *Citrobacter koseri*, *Staphylococcus aureus*, *Streptococcus pneumoniae*, *Micrococcus luteus,* and *Staphylococcus epidermis)* found in ground beef and trout fillets [52].

While many BC found in essential oils and plant extracts have antimicrobial properties that can prolong the shelf-life of MP, high doses can also induce pro-oxidant effects in meat matrices. It is important to carefully consider and optimize the amounts to be used to ensure their effectiveness without causing any unwanted effects.

### 4.6 Application and concentration of bioactive compounds in meat products

Bioactive compounds added to MP can prevent the formation of oxidized compounds that occur during processing, heat treatment, smoking, fermenting, or curing [[86], [87]], and can also help to control the deterioration of the meat by initially reducing the viable microbial population [67]. However, BC have limitations in their use due to their low stability, volatility, and high sensitivity to environmental factors. These compounds can also negatively affect the sensory properties of the food if not used in appropriate doses [30,76].

Despite these limitations, one major advantage of BC is that they are generally safe to use in food products. Extremely high concentrations would need to be ingested on a daily basis to cause any negative effects on human health [31,69]. Table 1 provides an overview of the use and dosage of various BCs and their effects on MP.

As demonstrated by Elhadi et al. [85], BC should be used in low doses in MP because they can negatively affect their sensory characteristics [53]. When 100 g/kg of moringa leaf powder was added to chicken burgers, people found the color, flavor, tenderness, and juiciness inferior to the control.

Alp & Aksu [3] observed that the efficiency of adding 500 ppm of nettle extract in ground meat is better than adding 250 ppm (Table 1). The applied dose of extract reduced LA but did not have a beneficial effect on the red color (a*), which caused product rejection. In another assay, Boruzi and Nour [84] used 0.5% walnut leaf powder to preserve refrigerated pork burgers (4 °C) for up to 15 days, with better results in appearance, color, flavor and general acceptability in comparison with 0.1% BHT.

Continuous research is required to develop the most efficient natural preservatives that can extend the shelf life of food, improve its sensory properties, ensure its safety for human consumption, and be environmentally friendly. The use of BC in meat preservation must also be studied continuously to achieve these goals. To ensure the reproducibility of results, it is necessary to report the processes required to obtain BC, the appropriate doses, and the different forms of use in various meat matrices.

## 5 Conclusions

Meat and MP have a pro-oxidant matrix that can result in the formation or ROS and RNS in spoilage and decreased shelf life. However, adding PE or EO with BC which contains various phenolic antioxidants, as a natural alternative to synthetic preservatives, has been shown to improve the shelf-life of meat and MP when used in doses ranging from 0.025 to 2.5% in combination with emerging packaging techniques. This suggests that BC can be a potential natural preservative for MP, which is not only effective but can also be environmentally friendly.

## 6 Future perspectives

The use of BC for meat preservation is an area of active research, and there are several promising future perspectives. One approach is to use natural compounds such as plant extracts, essential oils, and enzymes to extend the shelf life of meats. These compounds have antioxidant and antimicrobial properties that can inhibit the growth of spoilage and pathogenic microorganisms.

Another approach is the use of edible coatings and films made from natural materials such as chitosan, alginate, and starch, that can protect meat from microbial contamination and oxidation. These coatings can also improve the sensory properties of meat and increase its shelf life.

In addition, nanotechnology is being explored as a tool for improving the delivery and efficiency of BC. Nanoencapsulation can protect BC from degradation, and controlled release can ensure a sustained effect on microbial growth.

Overall, the use of BC for meat preservation holds great promise for extending the shelf life of meats, reducing food waste, and increasing the safety and quality of MP.

## Author contributions

Conceptualization, G.O.-A.; E.V.-B.-P. and A.T.P.-V.

Methodology, G.O.-A. and A.T.P.-V.

Validation, A.T.P.-V. and M.R.S.-C.

Investigation, G. O-A. and J.R.S.-G.

Writing – Original Draft Preparation, G. O-A.

Writing – Review & Editing, A.J.C.-C.; M.R.S.-C., A. S.-Z. and E.V–B–P.

Visualization, M.R.S.-C.

Supervision, A.J.C.-C.; E.V.-B.-P. and A.T.P.-V.

Project Administration, A.J.C.-C.; A.T.P–V.

Funding Acquisition, A.J.C.-C.; E.V.-B.-P. and A.T.P.-V.

## Declaration of competing interest

The authors declare that they have no known competing financial interests or personal relationships that could have appeared to influence the work reported in this paper.
